# Efficacy of a biofungicide based on *Trichoderma afroharzianum* CP 24–6 against cacao frosty pod rot under different doses and application timings

**DOI:** 10.3389/fpls.2026.1694820

**Published:** 2026-02-10

**Authors:** Wagner Meza-Maicelo, Anthony Cortez-Lázaro, Enistein R. Reyna-Rivera, Melissa I. Loja-Torres, Manuel Oliva-Cruz, Santos T. Leiva-Espinoza

**Affiliations:** 1 Facultad de Ingeniería y Ciencias Agrarias, Universidad Nacional Toribio Rodríguez de Mendoza de Amazonas, Chachapoyas, Amazonas, Peru; 2 Instituto de Investigación para el Desarrollo Sustentable de Ceja de Selva, Universidad Nacional Toribio Rodríguez de Mendoza de Amazonas, Chachapoyas, Amazonas, Peru

**Keywords:** artificial infection, biological control, endophyte, formulation, solid fermentation

## Abstract

During their development, cacao fruits are the primary target of frosty pod rot (Moniliophthora roreri), a disease that has affected cacao producers in Amazonas, Peru, for more than three decades. The objective of this study was to evaluate the biocontrol potential of two doses of Trichoderma afroharzianum CP 24–6 against artificial infection by M. roreri in cacao fruits at different developmental stages. For this purpose, a biofungicide was formulated using this native species, an endophyte from cacao agroecosystems in the region. After quality control, the biofungicide was applied at two doses (1 × 10^5^ and 1 × 10^6^ conidia/mL) and at three fruit developmental stages (20, 40, and 60 days), generating six treatments through the interaction of both factors. Incidence, external severity, and internal severity were evaluated under a completely randomized block design with a factorial arrangement. The results showed that the interaction between biofungicide dose and fruit age at the time of infection was a key determinant of the biocontrol efficacy exerted by T. afroharzianum. The dose of 1 × 10^6^ conidia/mL exhibited greater effectiveness, particularly in fruits infected at 60 days of development, whereas the 1 × 10^5^ conidia/mL dose was less effective at earlier stages. Application of the biofungicide delayed symptom expression and reduced both external severity and internal severity compared with untreated fruits, which showed higher susceptibility and more severe damage. These findings demonstrate the potential of T. afroharzianum CP 24–6 as a native biofungicide for the sustainable management of cacao frosty pod rot.

## 1 Introduction

Cacao (*Theobroma cacao* L.; Malvaceae), whose center of origin is located in the Upper Amazon region of South America (Díaz-Valderrama et al., 2020; Motamayor et al., 2002), has gained remarkable importance worldwide (Kongor et al., 2024). The beans of its fruit are in high demand globally, particularly in European and North American countries, where they constitute the main ingredient in the chocolate industry (Guevara-Viejó et al., 2024). This has turned cacao into a highly relevant commercial crop that sustains the livelihoods of five million smallholder farmers worldwide (Bandanaa et al., 2025; Lander et al., 2025).

However, the supply of cacao beans faces multiple limitations, including significant losses caused by pests and diseases, aging farms and trees, low plantation yields, and in some areas, the effects of climate change (Lahive et al., 2019). Fungal diseases represent a constant threat regardless of the production region (Marelli et al., 2019). Among them, frosty pod rot stands out as a highly invasive and endemic disease, caused by the phytopathogenic fungus *Moniliophthora roreri* (Cif.) Evans et al. (Basidiomycete, Marasmiaceae) (Tirado-Gallego et al., 2016).

This disease affects only cacao fruits at any developmental stage, causing both external and internal damage, which can lead to yield losses of up to 80% (Jiménez-Zapata et al., 2022; Krauss et al., 2010). Among the most common symptoms are fruit deformation, premature ripening, and the appearance of brown-colored lesions, while the characteristic sign is the presence of powdery spores covering these lesions, with no visible fruiting bodies, whose coloration becomes darker as they reach maturity (Díaz-Valderrama and Aime, 2016; Minio et al., 2023; Phillips-Mora et al., 2006).

In Peru, the disease was first reported in 1989 in the village of Quebrada Seca, Utcubamba province, in the Amazonas region (Díaz-Valderrama et al., 2020). Since then, it has become the main phytosanitary challenge for cacao cultivation (Leiva-Espinoza, 2022). Despite the restrictions caused by the presence of this disease, in 2016 the native cacao from the provinces of Bagua and Utcubamba, in the Amazonas region, obtained the designation of origin *Cacao Amazonas Peru*, a distinction that recognizes its unique characteristics, such as its ‘fine flavor’ quality, attributable to the geographical environment where it is produced (INDECOPI, 2024).

In response to this problem, multiple recommendations have been made for the control of frosty pod rot, starting with the weekly removal of diseased fruits, pruning, and timely harvesting of healthy fruits (Cubillos, 2024). The use of chemical fungicides has also been recommended, as they can reduce the damage caused by the disease and improve productivity (Bateman et al., 2005). However, their application poses risks to both human health and the environment (Deberdt et al., 2008; Ndoumbe-Nkeng et al., 2004), in addition to being a practice incompatible with the standards required for organic certification (Mockshell et al., 2025).

In this context, biological control emerges as an alternative to the use of chemical fungicides, since it employs living organisms or their derivatives to suppress the disease (Andrade-Hoyos et al., 2023; Guevara-Viejó et al., 2024). Within this type of control, multiple organisms have been used for the *in vivo* control of cacao frosty pod rot. For instance, Páez Martínez et al. (2024), reported high biocontrol efficacy when testing endophytic bacterial strains of *Bacillus* sp. Similarly, Krauss et al. (2003), evaluated isolates of *Clonostachys* sp. and *Trichoderma* sp., with the latter proving to be the most effective. Along the same lines, Leiva-Espinoza (2022), identified a high biocontrol potential in native strains of *Trichoderma* spp., reinforcing its importance as a biological control agent.

The genus *Trichoderma* (Hypocreaceae, Hypocreales, Ascomycota) comprises more than 500 recorded species (Zhao et al., 2025), many of which have been recognized as biological control agents, plant growth promoters, producers of enzymes and antibiotics, and as bioremediators capable of removing heavy metals (Saldaña-Mendoza et al., 2023). Their mechanisms of action as biological control agents include mycoparasitism, competition, and antibiosis (Ali Nusaibah and Musa, 2019). Their use, either individually or in consortium with other microorganisms, has been reported in multiple crops such as wheat (El-Sharkawy et al., 2025), tomato (Khairy et al., 2025), strawberry (Valfrè et al., 2025), soybean (Dos Santos et al., 2025), chickpea (Kumar et al., 2025), cotton (Javed et al., 2025) and cacao (Leiva-Espinoza, 2022).

For the mass production of *Trichoderma*, both solid and liquid formulations have been used (Sudha et al., 2024), due to its ability to easily propagate on various carbon- and nitrogen-rich substrates (Hasan, 2020). Among these, rice is one of the most commonly used substrates for this purpose (Naeimi et al., 2020), as it allows the production of viable propagules, such as conidia and chlamydospores, which are more resistant to desiccation than vegetative hyphae (Cumagun, 2014). Under this scenario, the objective was to evaluate the biocontrol potential of *Trichoderma afroharzianum* CP 24–6 against artificial infection by *Moniliophthora roreri* in cacao fruits at different developmental stages.

## 2 Material and methods

The initial work was carried out in the Plant Health Laboratory at the National University Toribio Rodríguez de Mendoza of Amazonas. As a starting point, a *Trichoderma afroharzianum*-based biofungicide broth, a species with high biocontrol potential *in-vitro* and *in-vivo* (Leiva-Espinoza, 2022), was prepared, which underwent quality control tests before conducting field pathogenicity trials. In these trials, the biofungicide was applied to cacao fruits previously infected at different developmental stages. The following details are provided:

### 2.1 Mass production of conidia

For the mass production of *Trichoderma* conidia, pre-cooked rice was used as the substrate, following the methodology described by Gómez-Ramírez et al. (2013) with some modifications. In this trial, the CP 24–6 strain of *Trichoderma afroharzianum*, which is part of the fungal collection of the Plant Health Research Laboratory at the National University Toribio Rodríguez de Mendoza of Amazonas (UNTRM), was used. This strain was cultured on Potato Dextrose Agar (PDA) at 28 ± 2°C for six days. After this period, all fungal mycelium was collected and transferred to a Falcon tube containing 10 mL of sterile water supplemented with 5 % (w/v) chloramphenicol. The suspension was homogenized and subsequently inoculated, using 5 mL of the suspension in two flasks containing pre-cooked rice, previously sterilized in a vertical autoclave at 121°C and 15 psi for 25 minutes. The flasks were incubated at 28°C in darkness for three days and then under a photoperiod (12 h light/12 h dark) until completing nine days, with daily shaking to ensure homogeneous colonization of the substrate.

To recover the *Trichoderma* conidia, the contents of both flasks were transferred to a 2 L container, and 500 mL of distilled water was added. Two washes were performed by manual agitation and decantation. The supernatants from the first and second washes were combined to obtain a conidial concentrate, which was stored at 4°C for 48 hours to allow sedimentation. Finally, the recovered sediment, composed of conidia, was placed in a container and dehydrated in an oven at 40°C for 21 hours.

### 2.2 Biofungicide formulation

A solid formulation was prepared by manually mixing 25 % dehydrated *T. afroharzianum* conidia, used as the active ingredient, with 75 % corn starch as an inert material, following the methodology proposed by Zhang and Yang (2015). The mixture was homogenized, and 300 mL of distilled water was subsequently added. The resulting suspension underwent a sedimentation process under refrigeration at 4°C for 48 hours. After this period, the obtained sediments were dehydrated in an oven at 40°C for 13 hours. Finally, the dry material was manually ground, packaged in hermetic bags, and stored under refrigeration at 4°C until used in field applications.

### 2.3 Quality control

In this part of the process, quality parameters were evaluated, including conidial count, quantification of Colony Forming Units (CFU), purity, conidial germination percentage, pH, and moisture content. The following details are provided:

To perform the conidial count, a suspension was prepared by mixing 1 g of the formulation with 100 mL of sterile water, agitating the mixture for 30 seconds using a vortex. Subsequently, a 1:10 dilution was made, from which a 10 μL aliquot was taken with a micropipette and deposited in the central quadrant of a Neubauer chamber for counting (Gómez-Ramírez et al., 2013). The conidial dose per gram of formulation was calculated using the formula described by Reyes-Figueroa et al. (2016). For pH measurement, a Thermo Scientific benchtop pH meter (Orion Star™ A215) was used, with the electrode immersed in the initial suspension (Beretta et al., 2014), following the corresponding protocol.

For the quantification of Colony Forming Units (CFU) and the determination of purity percentage, serial dilutions were performed until reaching concentrations of 10^-7^, 10^-6^, and 10^-5^. From each dilution, 200 μL were inoculated in the center of Petri dishes containing PDA medium, both supplemented and unsupplemented with chloramphenicol at 0.05 µg/mL. Subsequently, using sterile glass beads, the inoculum was evenly distributed over the surface of the medium. The plates were sealed and incubated at room temperature for 48 hours. After this period, colonies were visually counted, and the results were recorded as CFU/g (Naeimi et al., 2020). To determine the purity percentage, the number of CFU corresponding to both contaminants and *T. afroharzianum* was recorded and averaged (Gómez-Ramírez et al., 2013).

For the determination of conidial germination percentage, serial dilutions were performed until reaching a final concentration of 10^-^². From this dilution, 150 μL were inoculated onto plates containing bacteriological agar, which were incubated at room temperature for 20 hours. After this period, a 1 cm² agar fragment was extracted using a scalpel and transferred to a microscope slide. A drop of lactophenol blue was added, and a coverslip was placed on top. The observation and counting of germinated and non-germinated conidia were performed using a light microscope (ZEISS Primostar 3 iLED) at 60× magnification (Gómez-Ramírez et al., 2013; Naeimi et al., 2020). Finally, for the determination of moisture content, a 1 g sample of the formulation was weighed and dried in an oven at 120 °C for 24 hours, with the procedure performed in quintuplicate (Radulovich, 2008).

### 2.4 Field pathogenicity trial

**Figure 1:**
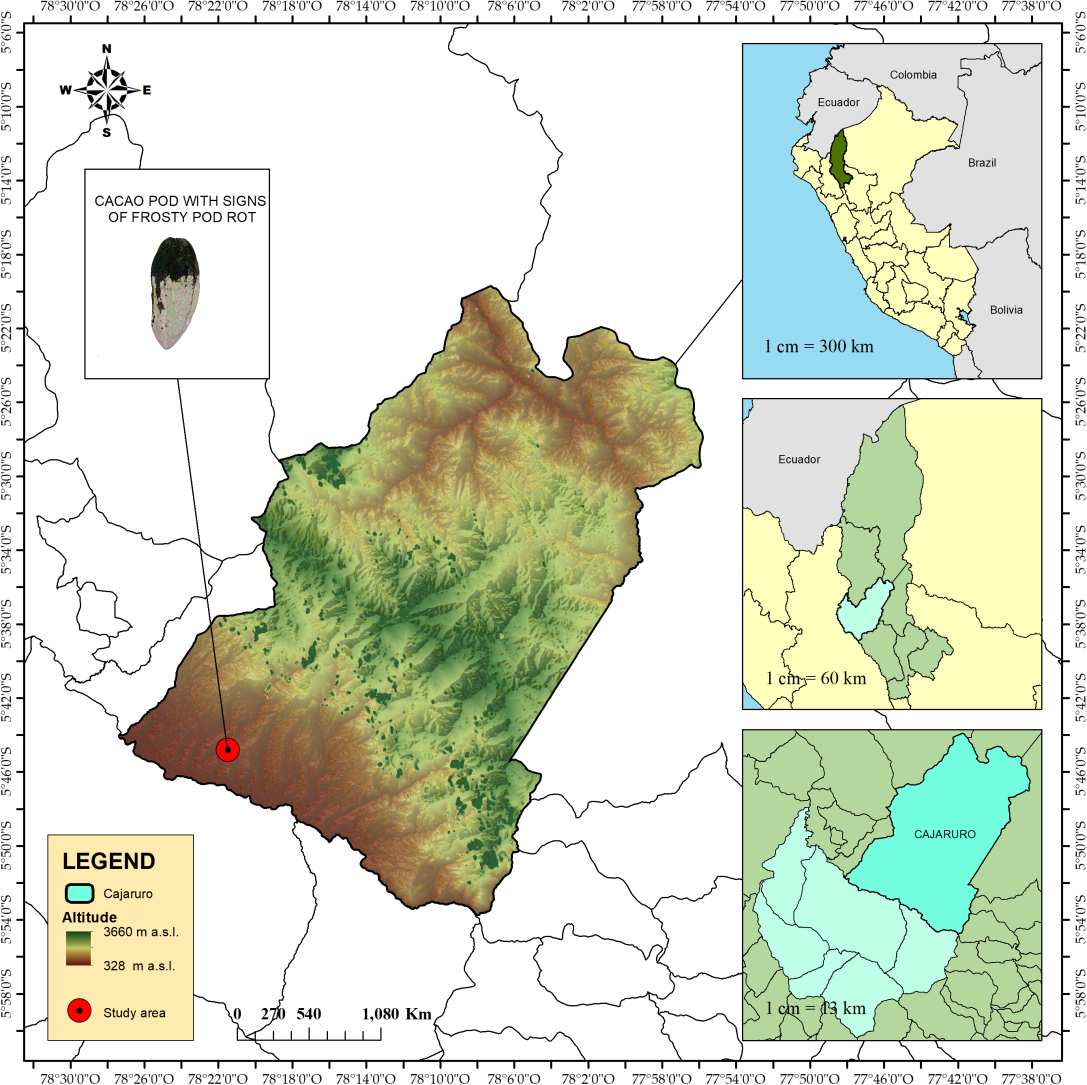
Location of the field experimental trial.

The field trial was conducted in a ten-year-old *Indes 31* cacao plantation (Oliva and Maicelo Quintana, 2020) with low incidence of cacao frosty pod rot. This plantation is part of the clonal garden of fine-flavor native cacao, belonging to the Research Institute for Sustainable Development of Ceja de Selva (INDES-CES) at UNTRM, located in the district of Cajaruro, Utcubamba province, at an altitude of 700 m above sea level, with geographic coordinates 5° 44′ 47.638″ S and 78° 21′ 28.908″ W (**Figure 1**).

Prior to the application of treatments, maintenance pruning of the cacao plantation was carried out, which involved the removal of approximately 15 % of the leaf area to stimulate the production of a higher number of flowers per plant (Vera-Chang et al., 2024). The trial was adapted to a completely randomized block design (CRBD) with a 2 × 3 factorial arrangement, evaluating two factors: conidial dose (levels: 1 × 10^5^ and 1 × 10^6^ conidia/mL) and fruit age at the time of infection (levels: 20, 40, and 60 days), generating six treatments: T1 (1 × 10^5^: 20), T2 (1 × 10^5^: 40), T3 (1 × 10^5^: 60), T4 (1 × 10^6^: 20), T5 (1 × 10^6^: 40) and T6 (1 × 10^6^: 60), with five replications each. Finally, a control was included for each fruit age at infection (C20, C40 and C60). Treatments were randomly assigned within each block.

Subsequently, flowering in the cacao plants was monitored, after which flowers that underwent natural pollination were isolated. This isolation was carried out using 100 mL Falcon tubes until the initial fruit set, after which the isolation condition was replaced with a polypropylene bag to ensure hermetic sealing and prevent external contamination. Treatments were applied at three time points: when fruits reached 20, 40, and 60 days post-fruit set. In each case, fruits were kept covered and/or isolated with plastic bags and labeled according to the proposed experimental design (Alejandria- Tapia, 2021). At each time point, a corresponding control was included, consisting of fruits isolated and artificially infected at the same age as the other treatments.

Next, the biofungicide was applied at the corresponding concentrations and time points according to the planned treatments. The application was directed to the previously identified and isolated cacao fruits using hand sprayers, with the spray applied locally. Applications were carried out every 20 days after the first application (according to the starting time for each treatment), using volumes of 1, 2, and 3 mL of biofungicide broth for fruits at 20, 40, and 60 days of development, respectively, adjusting the volume proportionally to the size of the fruit (Calle-Cheje et al., 2023; Cuenca Sedamanos et al., 2022).

Following the application of the biofungicide broth, the fruits were inoculated with *Moniliophthora roreri* to induce infection and the development of frosty pod rot, starting with the fruits corresponding to the treatment whose first application was carried out at 20 days of age, following the protocol of Leandro Muñoz et al. (2025) with some modifications. For this purpose, small holes were made in the protective bags using a needle to insert the sprayer nozzle and allow direct application onto the fruits. Inoculation was performed using a sterilized sprayer, applying 1, 2, and 3 mL of inoculum once (1 × 10^5^ conidia/mL), according to fruit age and the timing of the first application (20, 40, and 60 days, respectively).

External severity (ES) was assessed every 20 days, starting 20 days after infection, following the methodology of Sánchez-López (1983), using a scale from 0 to 5, where 0 indicates a healthy fruit and 5 a completely mummified fruit. The values obtained were used to calculate the Area Under the Disease Progress Curve (AUDPC), according to Li (2024). Internal severity (IS) was determined at harvest and expressed as the percentage of necrosis of internal tissue, using a scale ranging from 0 % to over 80 %. At the same time, disease incidence was calculated using the formula of Krauss and Soberanis (2002): Incidence= (Infected fruits/Total evaluated fruits) *100. For this calculation, fruits without visible infection symptoms during external severity evaluations were also considered. Finally, the percentage of biocontrol efficacy for each treatment was estimated from the IS data, following Abbott (1925).

### 2.5 Statistical analysis

The assumptions of normality and homogeneity of variances were verified using the Shapiro-Wilk and Bartlett tests, respectively. Subsequently, an ANOVA was performed followed by Tukey’s test (p ≤ 0.05) for multiple comparisons, using the R statistical software (R Core Team, 2025).

## 3 Results

### 3.1 Analysis of quality parameters and formulation

During fermentation, *T. afroharzianum* CP 24–6 uniformly colonized the rice substrate by the ninth day of incubation. Initially, the substrate appeared whitish, gradually turning green over the course of the days. After formulation, quality control revealed a dose of 1.1 × 10¹^0^ conidia/g. Additionally, 3.1 × 10^9^ CFU/g were quantified. No contaminants were detected, resulting in 100 % purity. The product had a pH of 5.8, a germination rate of 96.02 %, and a moisture content of 4.5 %.

### 3.2 Evaluation of biocontrol potential

During the evaluation period, spanning from infection to post–fruit set harvest, the time to symptom appearance and their external expression were recorded in fruits infected at different developmental stages. In fruits infected at early stages of development (20 and 40 days), the first symptoms appeared approximately 20 days after infection, mainly characterized by yellowing and visible deformations of the pods. In contrast, in fruits infected at a more advanced stage (60 days), symptom appearance was delayed, being observed from 40 days after infection and initially expressed as small brown spots that progressively increased over time. The ES values recorded throughout the evaluation period were integrated to calculate the AUDPC, allowing comparison of the effects of the different doses applied within each infection age. For all three ages evaluated, the dose of 1 × 10^6^ conidia/mL resulted in significantly lower AUDPC values than the dose of 1 × 10^5^ conidia/mL and the corresponding control (**Table 1**).

**Table 1:** Mean values (± standard deviation) of the area under the disease progress curve (AUDPC), calculated from external severity, in cacao fruits infected at 20, 40, and 60 days of development and treated with *Trichoderma afroharzianum* at doses of 1 × 10^5^ and 1 × 10^6^ conidia/mL, in comparison with the control treatment.

Doses (conidia/mL)	Cacao pod ages (days)		
	20	40	60
1 × 10^5^	210.8 ± 2.59 (b*)	66.4 ± 2.88 (b*)	20.6 ± 1.34 (b*)
1 × 10^6^	61.6 ± 0.89 (c*)	19.6 ± 1.67 (c*)	9.2 ± 1.79 (c*)
control	360.4 ± 2.61 (a*)	234.8 ± 2.68 (a*)	149.6 ± 1.82 (a*)

Different letters within each column indicate statistically significant differences among doses according to Tukey’s multiple comparison test (p ≤ 0.05). The use of an asterisk (*) indicates a statistically significant difference at p ≤ 0.05.

At harvest, the final assessment of ES was performed, and IS was evaluated at the same time. A significant interaction between the dose of *T. afroharzianum* and fruit age at the time of infection was detected (α < 0.05). *Post hoc* comparisons indicated that the effect of the biocontrol agent on ES and IS depended on fruit age. In addition, a very strong positive correlation was observed between ES and IS (r = 0.98), indicating that higher visible severity was associated with greater internal damage.

**Figure 2:**
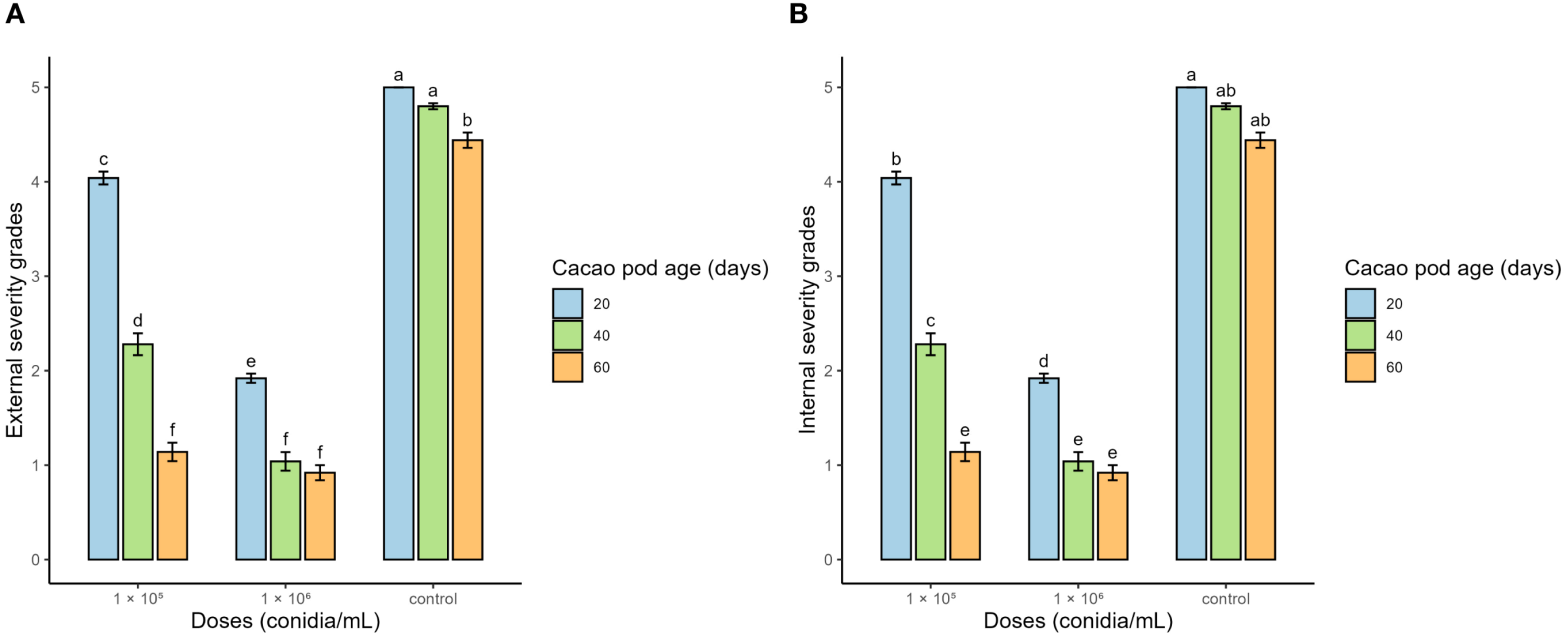
Degrees of external severity **(A)** and internal severity **(B)** of cacao moniliasis in fruits infected at 20, 40, and 60 days of age, in response to the application of *Trichoderma afroharzianum* at doses of 1 × 10^5^ and 1 × 10^6^ conidia/mL, as well as the control treatment. Bars represent the mean ± standard deviation. Different letters above the bars indicate statistically significant differences among treatments according to Tukey’s multiple comparison test (p ≤ 0.05).

**Figure 3:**
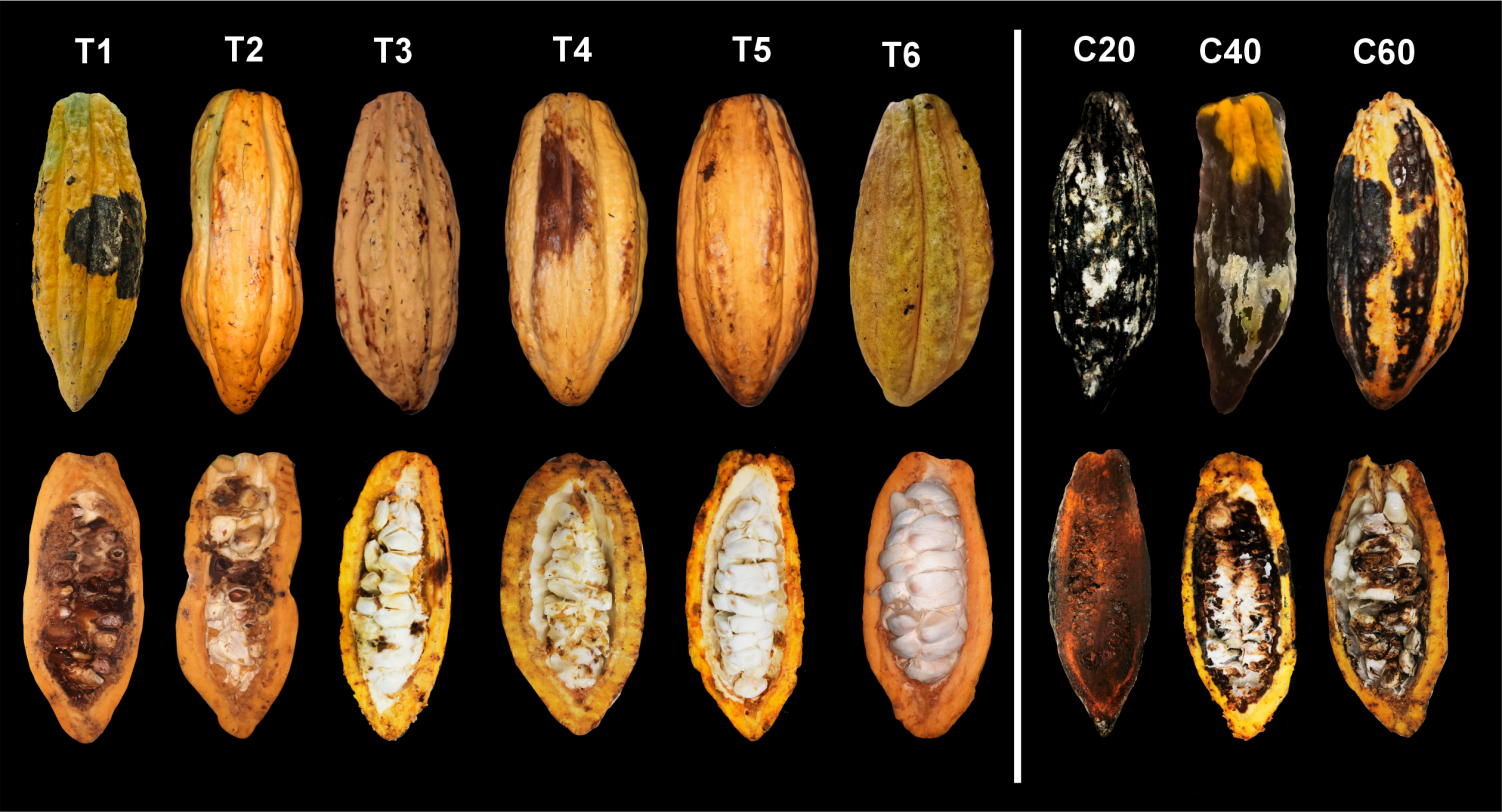
Representative fruits showing external and internal symptoms of cacao moniliasis following artificial inoculation with *Moniliophthora roreri* under different biocontrol treatments. Treatments T1, T2, and T3 correspond to the application of *Trichoderma afroharzianum* at a dose of 1 × 10^5^ conidia/mL in fruits infected at 20, 40, and 60 days, respectively, whereas treatments T4, T5, and T6 correspond to the application of 1 × 10^6^ conidia/mL in fruits infected at 20, 40, and 60 days, respectively. Treatments C20, C40, and C60 represent untreated control fruits infected at 20, 40, and 60 days, respectively. Upper images show external symptom expression, and lower images show internal tissue damage at harvest.

The significantly lowest degrees of SE and SI were recorded in the treatments with 1 × 10^6^ conidia/mL applied to fruits infected at 40 (T5) and 60 days (T6), as well as in the treatment with 1 × 10^5^ conidia/mL applied at 60 days (T3). In these treatments, SE degrees ranged between 0.92 and 1.14 (**Figure 2A**), associated with mild symptoms such as small brown spots and exudation (**Figure 3**), whereas SI degrees ranged between 1.22 and 1.48 (**Figure 2B**), corresponding to 24%–29.6% of necrotic internal tissue (**Figure 3**). In contrast, the control group and the treatment with 1 × 10^5^ conidia/mL applied at 20 days showed the highest severity degrees, with SE degrees between 4.04 and 5 (**Figure 2A**) and SI degrees between 4.6 and 5 (**Figure 2B**), characterized by extensive necrosis, abundant sporulation, and completely mummified fruits, representing 92%–100% of necrotic internal tissue (**Figure 3**).

At the end of the ES and IS assessments, disease incidence was recorded, considering all fruits showing any type of symptom as infected. This included fruits that, despite being asymptomatic in previous external severity evaluations, exhibited internal symptoms at harvest. Based on this record, statistical analysis (ANOVA) showed no significant differences between treatments for disease incidence (p > 0.05).

**Figure 4:**
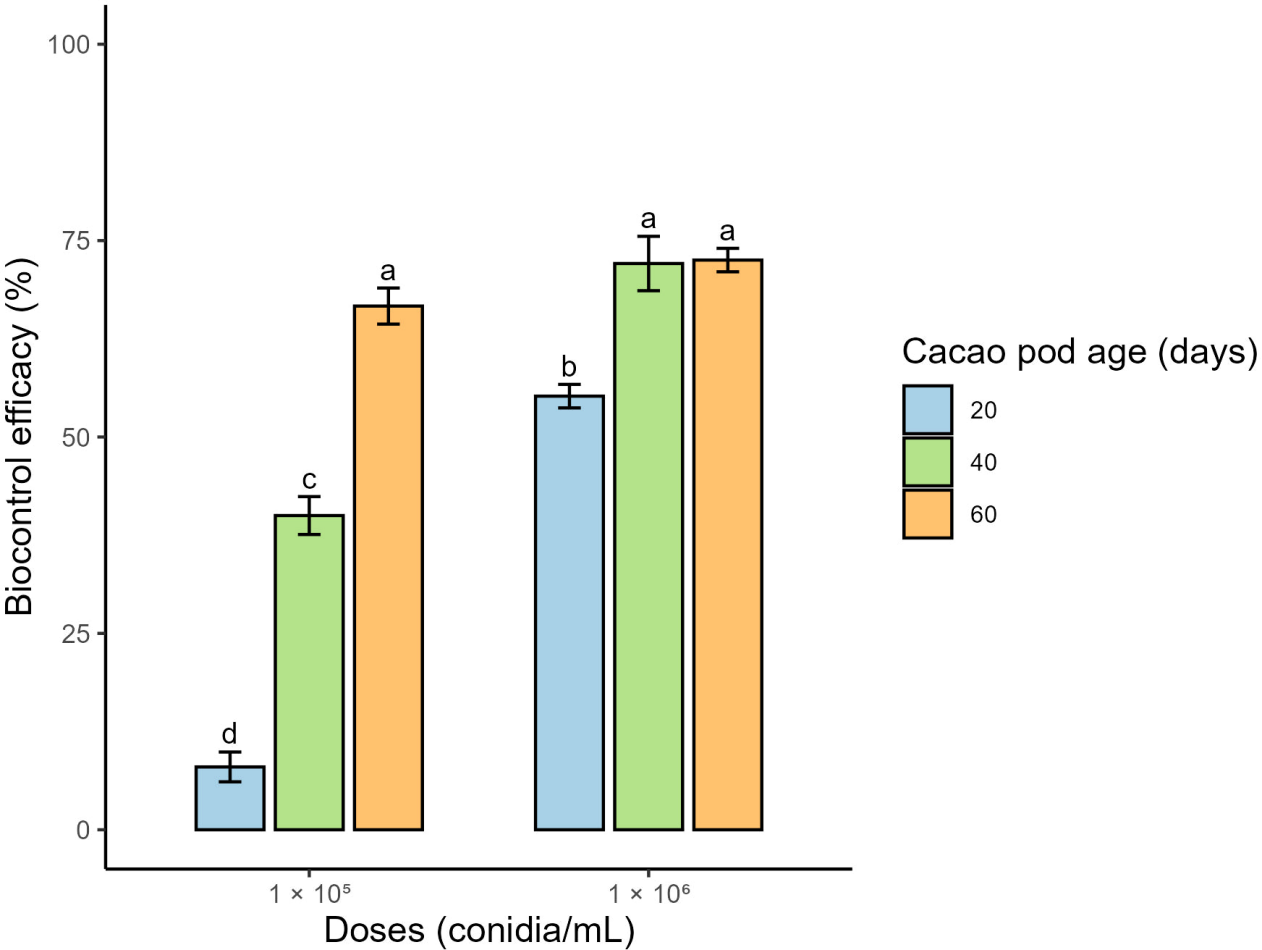
Biocontrol efficacy (%) of the biofungicide formulated with *Trichoderma afroharzianum* CP 24–6 against cacao frosty pod rot in fruits infected at 20, 40, and 60 days of age and treated with doses of 1 × 10^5^ and 1 × 10^6^ conidia/mL. Bars represent the mean ± standard deviation. Different letters above the bars indicate statistically significant differences among treatments according to Tukey’s multiple comparison test (p ≤ 0.05). Efficacy was calculated based on the percentage reduction of internal severity relative to the untreated control.

To determine treatment efficacy, internal severity (IS) grades were converted to percentages. The results showed that the application of 1 × 10^6^ conidia/mL to fruits infected at 40 (T5) and 60 days (T6), as well as the application of 1 × 10^5^ conidia/mL to fruits infected at 60 days (T3), achieved significantly higher efficacy percentages compared with the remaining treatments, with values of 72.1%, 72.5%, and 66.7%, respectively (**Figure 4**).

## 4 Discussion

This study developed an approach for controlling frosty pod rot through the application of a biofungicide based on *Trichoderma afroharzianum* CP 24–6 on fruits of native fine-aroma cacao, ecotype Indes 31, infected at different ages in Amazonas, Peru. Initial production of *T. afroharzianum* conidia was carried out through solid-state fermentation using rice as the substrate. For the formulation, the conidia were combined with corn starch as an inert material, and the formulated biofungicide was subsequently subjected to a quality control process. Disease incidence and ES were evaluated at 20-day intervals after infection, while IS of frosty pod rot was assessed at harvest.

For *M. roreri* to initiate infection in cacao fruits, its conidia must germinate and penetrate directly through the epidermis or via stomata, colonizing the tissue intercellularly without inducing an antagonistic response, such as cell necrosis (Evans, 1981, Evans, 2016a; Suárez, 1972). Although young fruits are more susceptible during the first 45 days of development, they can be infected at any age, although susceptibility decreases with maturation (Evans, 1981). After infection, the fungus employs stealthy mechanisms to evade plant defenses and enters a biotrophic or asymptomatic phase that can extend up to 60 days, during which it develops internally without producing visible symptoms (Meinhardt et al., 2014). At this stage, the pathogen induces the accumulation of aqueous exudates resulting from enzymatic tissue maceration, and as the necrotrophic phase begins, chlorotic spots appear, expanding and merging to form necrotic brown lesions on the fruit surface (Da Silva Estrela Junior et al., 2023; Leach et al., 2002). Within 4 to 5 days, these lesions become covered with powdery conidia, lacking a fruiting body, which darken as the spores mature (Díaz-Valderrama et al., 2020; Evans, 1981, Evans, 2016).

In the present study, fruits that did not receive biofungicide application and were infected at early ages exhibited an earlier expression of disease symptoms compared with fruits infected at later ages. Disease progression was characterized by the gradual development of necrotic lesions that tended to coalesce, followed by pathogen sporulation, which ultimately led to the formation of mummified fruits at harvest. This pattern of frosty pod rot development is consistent with that reported by Bailey et al (2013), who identified fruit malformations as the predominant symptom around 30 days after infection. Similarly, Phillips Mora (1986) reported a higher frequency of exudation and gibbosity formation at approximately 23 days, whereas premature yellowing and necrotic lesions occurred less frequently, although sporulation persisted until harvest. Likewise, Evans (1981) and Bailey et al. (2013), reported the onset of sporulation around 60 days after infection.

The application of the biofungicide was reflected in the AUDPC values, which indicated a significant reduction in disease advancement compared with fruits that did not receive treatment, in which disease progression occurred without restriction. In particular, application of the biofungicide at a dose of 1 × 10^6^ conidia/mL significantly reduced disease advancement, whereas the control treatment exhibited the highest AUDPC values. These results are consistent with those reported by Villamil-Carvajal et al. (2015), who also observed the highest AUDPC values in untreated fruits. Although the benefits of using biological control agents are widely recognized, their effectiveness largely depends on detailed knowledge of their mechanisms of action (Villavicencio-Vásquez et al., 2025). In the present study, the biofungicide was applied prior to artificial infection, following a preventive approach, which is supported by the fact that species of the genus *Trichoderma* are more effective when they are established before the pathogen encounters favorable conditions for colonization (Ferreira and Musumeci, 2021).

In addition to the preventive application, other factors explaining the observed effectiveness include application frequency and inoculum dose. Under high disease pressure conditions, such as artificial infections, Hanada et al. (2009) indicate that frequent application of the biocontrol agent during fruit development, combined with high concentrations, is necessary to achieve effective pathogen inhibition. This is further supported by the use of the native strain *T. afroharzianum* CP 24-6, which has demonstrated remarkable biocontrol potential both *in-vitro* and *in-vivo* (Leiva-Espinoza et al., 2020; Leiva-Espinoza, 2022). This native strain likely contributed significantly to disease suppression and the reduction of symptom severity. In this regard, Ferreira and Musumeci (2021) highlight that the use of native species represents an appropriate strategy to avoid the risks associated with introducing exotic organisms, as they are adapted to the specific environmental conditions, such as temperature, humidity, and nutrient availability.

The evaluations conducted throughout fruit development revealed high levels of disease incidence, defined as the proportion of fruits exhibiting at least one symptom relative to the total number of fruits assessed (Ngo Bieng et al., 2017). This elevated incidence limited the detection of significant differences among treatments. Similar results were reported by Villamil-Carvajal et al. (2015), who observed 100% incidence of frosty pod rot in all treatments from the third week onward, regardless of the application of antagonistic microorganisms. Likewise, Paredes-Espinosa (2018) recorded incidence values ranging from 90% to 100% following artificial inoculations. In contrast, Leiva-Espinoza (2022) reported substantially lower incidence levels under natural infection conditions, with values ranging from 29% to 49% when *T. afroharzianum* CP 24–6 was applied, whereas incidence in untreated fruits ranged from 62% to 71%. Collectively, these findings indicate that artificial infection, together with environmental conditions favorable for pathogen germination and establishment, plays a decisive role in the high incidence levels observed.

However, incidence alone does not provide a complete view of the damage a disease can cause, and it is therefore complemented by severity indicators. This parameter allows for estimating the affected area of the tissue, expressed in absolute values or percentages, facilitating a more precise characterization of disease progression (Madden and Hughes, 1995). According to González et al. (2011), ES corresponds to the fruit’s appearance and visible signs of the pathogen, as it measures the surface damage caused by the fungus and its ability to produce propagules. In contrast, IS is expressed as the percentage of necrosis observed inside the fruit after making a longitudinal cut.

The ES and IS results showed a positive correlation between both variables, indicating that greater visible severity on the fruit was associated with greater internal damage. This pattern is consistent with the findings of Phillips Mora (1986), who also reported a positive correlation between these parameters. Furthermore, the severity levels recorded in the final evaluation indicated that the effectiveness of the biological control agent was influenced by both fruit age at the time of infection and the conidial concentration in the biofungicide. In general, biofungicide application in fruits infected at later ages was associated with less severe symptoms and signs, compared with untreated fruits or fruits treated and infected at earlier ages, in which disease severity was higher. These results are consistent with those reported by Villamil-Carvajal et al. (2015), who observed lower severity levels following the application of a *T. harzianum* strain compared with untreated fruits. Similarly, Chuquibala-Checan (2019) reported reduced severity at higher concentrations of the CP 24–6 strain of *Trichoderma afroharzianum*, indicating a dose-dependent response. Overall, these findings suggest that the use of native *Trichoderma* strains, combined with the application of optimal concentrations and consideration of fruit age, constitutes a promising strategy to enhance the effectiveness of biological control in fruits infected by *M. roreri*.

Finally, IS was considered the most representative parameter for evaluating biocontrol efficacy, as it more accurately reflects the damage the pathogen can cause to the commercial product (Phillips-Mora et al., 2005). The results showed that the fruit’s age at the time of infection was a decisive factor in the biocontrol agent’s performance, with lower efficacy observed in treatments applied at 20 days of development (T1 andT4). Similarly, the applied dose had a marked effect on the response obtained; in particular, the application of 1 × 10^6^ conidia/mL (T5) was significantly more effective than 1 × 10^5^ conidia/mL (T2) in fruits of the same age. This difference was reflected in the reduction of internal damage, which was 40 % with the lower dose, compared to 72.1 % achieved with the higher dose. In comparison, Leiva-Espinoza (2022) reported an efficacy of 59 % using the same strain (*T. afroharzianum* CP 24-6), albeit under natural infection conditions. These results suggest that applying the biofungicide at an appropriate dose can significantly enhance biocontrol efficacy.

## 5 Conclusions

This study demonstrated that the interaction between the biofungicide dose and the infection age is a decisive and significant factor in the biocontrol efficacy of *Trichoderma afroharzianum* CP 24-6, a native species from the cacao agroecosystem of Amazonas, Peru. The high dose (1 × 10^6^ conidia/mL) showed greater efficacy, particularly in fruits infected at 60 days, while the low dose (1 × 10^5^ conidia/mL) was less effective at early developmental stages. The application of the biofungicide not only delayed the appearance of initial symptoms but also reduced both external and internal severity of the disease. In contrast, in the control group, fruits infected at early stages (20 and 40 days) and without biofungicide application were more susceptible and showed severe damage. On the other hand, in fruits infected at a late age (60 days), the onset of the first symptoms was delayed. However, infection, its progression, and the damage caused by the disease were inevitable.

## Data Availability

The data are available and can be accessed at the following GitHub repository: https://github.com/WAHGNERM2002/FROSTY-POD-ROT.
